# Screening Intervals for Diabetic Retinopathy and Implications for Care

**DOI:** 10.1007/s11892-017-0928-6

**Published:** 2017-09-05

**Authors:** Peter H. Scanlon

**Affiliations:** 1 Gloucestershire Retinal Research Group, Gloucestershire Hospitals NHS Foundation Trust, Office above Oakley Ward, Cheltenham General Hospital, Sandford Road, Cheltenham, GL53 7AN UK; 2 Oxford University Hospitals NHS Foundation Trust, John Radcliffe Hospital, Headley Way, Headington, Oxford, OX3 9DU UK

**Keywords:** Retinopathy, Retinal screening, Imaging, Screening interval, Sight-threatening diabetic retinopathy, Visual loss

## Abstract

**Purpose of Review:**

The purpose of this study is to review the evidence that lower risk groups who could safely be screened less frequently for sight-threatening diabetic retinopathy (DR) than annually.

**Recent Findings:**

Data have demonstrated that people with no DR in either eye are at a low risk of progression to sight-threatening DR over a 2-year period (event rate 4.8 per 1000 person years), irrespective of whether the screening method is one-field non-mydriatic or two-field mydriatic digital photography. Low risk has been defined as no retinopathy on two consecutive screening episodes or no retinopathy on one screening episode combined with risk factor data.

**Summary:**

The risk of an extension to 2 years is less than 5 per 1000 person years in a population with a national screening programme, and the general standard of diabetes care is relatively good, whether low risk is defined as no retinopathy on two consecutive screening episodes or no retinopathy on one screening episode combined with other risk factor data. The definition used in different populations is likely to depend on the availability of data.

## Introduction

In the UK, the Office for National Statistics publishes an interesting graph of life expectancy over the last two centuries [1], and this has demonstrated that life expectancy has doubled from 40.2 years for men and 42.2 years for women to 79 and 82.8, respectively. Life expectancy has risen around the world and, although there are still differences between countries, even countries with lower levels of life expectancy have also almost doubled their life expectancy during the last two centuries [2].

The increased life expectancy has led to a change in the nature of the diseases we are treating. There are many more patients in older age groups with chronic diseases such as diabetes and cancer. There is an epidemic of diabetes [3, 4] around the world, which has led to an epidemic of diabetic retinopathy (DR) [5].

Various DR screening programmes have been set up around the world. Population-based screening programmes tend to be more successful in countries with state-run nationalised health systems because of the infrastructure and finance that is in place to support these programmes. Early pioneering work on screening for DR was conducted in Iceland [6, 7] and Sweden [8], and this was later introduced in the UK [9–11], in other European countries [12–14] and in Singapore [15]. In the USA and Australia, screening programmes have tended to be in indigenous populations [16–18], linked to diabetes clinics [19], in Veterans Administration Healthcare systems [20], or in research programmes [21]. There has also been interest in the development of screening programmes for DR in India [22], China [23], and South America [24]. In England, the epidemic of diabetes has proved to be a problem for the screening programme because of the increasing numbers each year. When screening was introduced in England in 2003, it was believed that there were approximately 1.4 million people with diabetes who needed to be screened, and this number has risen to 2.6 million.

The annual report [25] of National Health Service (NHS) screening programmes in England 2015–2016 reported that there were 2.59 million people with diabetes offered screening with 2.14 million being screened (an uptake of 82.8%) with urgent referrals for proliferative DR of 7593 and routine referrals of 52,597. Routine referrals are either for moderate to severe non-proliferative DR or signs of maculopathy using two-dimensional markers. The rate of referable retinopathy per 100,000 screened was 2807. In the same period (2015–2016), the largest number of new registrations of people with diabetes was recorded—326,587.

In the first and second rounds of screening, the referable retinopathy rate was considerably higher than in subsequent rounds of screening. For example, in the Gloucestershire screening programme, the referable rate of retinopathy was 11.2% in the first round of screening in 1998–2000 [26]. In addition, in populations who are screened for diabetes [27–29], which is becoming more common, the rate of any DR is much lower (7.6, 6.8, and 9%) than the prevalence in a known population of people with diabetes. In a review of 35 studies by Yau [30], the overall prevalence of any DR was 34.6% (95% confidence interval (CI) 34.5–34.8), 6.96% (6.87–7.04) for proliferative DR, 6.81% (6.74–6.89) for diabetic macular oedema, and 10.2% (10.1–10.3) for vision-threatening DR.

### Purpose of This Review

These factors have all led to the need to re-evaluate the need for annual screening for the whole population of people with diabetes and ascertain whether there are lower risk groups who could safely be seen less frequently.

## Methods

The author has conducted an on-going literature review since March 2000 utilising Zetoc [31] which provides access to over 30,000 journals and more than 52 million article citations and conference papers through the British Library’s electronic table of contents**.**

The following subject title keywords are used: 

‘retinopathy’, ‘digital’ and ‘imaging’ and ‘eye’, ‘digital’ and ‘imaging’ and ‘ophthalm’, ‘digital’ and ‘imaging’ and ‘diabet’, ‘laser’ and ‘eye’, ‘laser’ and ‘ophthalm’, ‘laser’ and ‘diabet’, ‘visual’ and ‘acuity’, ‘visual’ and ‘impairment’, ‘blindness’ and ‘diabet’, ‘diabetic’ and ‘screening’, ‘uptake’ and ‘screening’ and ‘diabet’ in title, ‘attendance’ and ‘screening’ and ‘diabet’, and ‘vitrectomy’ and ‘diabet’, and ‘diabet’ and ‘screening’ and ‘interval’ in title.

The contents’ page lists of 27 journals, considered to be those most likely to publish articles relevant to this topic, are also reviewed each month. Articles of interest identified with this search strategy were sourced from the local NHS Trust library or online from electronic journal resources.

## Results

I have identified 26 articles relevant to the purpose of this review. Most of these articles come from established national or regional screening programmes with good population coverage and a relatively high standard of diabetes care, and so these findings may not be applicable to areas where the standard of diabetes care is not as high or where screening is not established.

A. Evaluation of real-world screening programmes

1. In 2003, Younis [32, 33] reported the incidence of sight-threatening retinopathy in patients with both type 1 and type 2 diabetes in the Liverpool Diabetic Eye Study.In type 1 diabetes [32], 501 patients underwent 2742 screen events. Cumulative incidence of sight-threatening DR in 305 patients without baseline retinopathy was 0.3% (95% CI 0.0–0.9) at 1 year, rising to 3.9% (1.4–5.4) at 5 years. The study concluded that screening at 2–3-year intervals, rather than annually, for patients without retinopathy in type 1 diabetes is feasible because of the low risk of progression to sight-threatening DR.

In type 2 diabetes [33], 4770 patients underwent 20,570 screening events. Yearly incidence of sight-threatening DR in 3743 patients without retinopathy at baseline was 0.3% (95% CI 0.1–0.5) in the first year, rising to 1.8% (1.2–2.5) in the fifth year; cumulative incidence at 5 years was 3.9% (2.8–5.0). The study concluded that a 3-year screening interval could be safely adopted for patients with no retinopathy.

2. In 2007, Olafsdottir [34] reported that 296 patients with diabetes in Iceland who had no DR in 1994/1995 were followed with biennial eye examinations until they developed any retinopathy when they were reviewed with annual eye examinations. Over the 10-year period, 172 did not develop any retinopathy, 96 developed mild non-proliferative DR, 6 developed clinically significant macular oedema, 23 developed moderate to severe non-proliferative DR, and 4 developed proliferative DR. All those who developed macular edema or proliferative DR had already been diagnosed with mild non-proliferative DR and entered annual screening before progressing to this level at a subsequent screening event. They concluded that biennials screening for those without retinopathy was safe.
3. In 2009, Misra et al. [35] analysed the results of 63,622 screening episodes among 20,788 people, 16,094 (25%) identified any retinopathy, 3136 (4.9%) identified referable retinopathy, and 384 (0.60%) identified sight-threatening DR (defined as proliferative DR or treatable maculopathy). They found that, compared with screening intervals of 12–18 months, screening intervals of 19–24 months were not associated with an increased risk of referable retinopathy [adjusted odds ratio 0.93, 94% CI 0.82–1.05].
4. In 2009, Soto-Pedre [36] reported on 286 patients from northern Spain with diabetes free of DR and 144 patients with mild non-proliferative DR at baseline who had been screened with one-field non-mydriatic photography. For the 286 patients free of DR, the probability of remaining free of sight-threatening DR was 97% (95% confidence interval [CI] 94–99%) at the end of the fourth year.
5. In 2011, Agardh [37] reported the experience of adopting 3-year screening intervals for sight-threatening retinal vascular lesions in subjects with type 2 diabetes without retinopathy in Sweden. 1691 subjects with type 2 diabetes and no detectable retinopathy in two 50° red-free fundus photographs were scheduled for follow-up with photography 3 years later. Age at diabetes diagnosis was 60 ± 12 years, and known duration of diabetes was 6 ± 6 years. Treatment consisted of diet only (26%), oral agents (54%), and oral agents and/or insulin (20%). Glycated hemoglobin (HbA1c) was 6.4 ± 1.5%. Of the 1322 subjects available for follow-up, 73% were still without retinopathy after 3 years, and 28% had developed mild or moderate retinopathy, but none developed severe non-proliferative or proliferative retinopathy. Macular edema requiring laser coagulation occurred in only one eye. The study concluded that 3-year retinal screening intervals can be recommended in subjects with type 2 diabetes and no retinopathy.
6. In 2013, Scanlon [38] reported that the risk of progression of DR is significantly higher for those with background DR in both eyes than those with background retinopathy in only one or in neither eye. Prior to these low-risk groups were generally regarded to be those people with no DR in either eye.
7. In 2013, Porta [39] published the clinical characteristics influencing screening intervals for DR. The cumulative incidence, time of development, and relative risk of developing referable retinopathy over 6 years following a negative screening for DR were calculated in 4320 patients, stratified according to age at onset of diabetes (<30 or ≥30 years), being on insulin treatment at the time of screening and known duration of diabetes (<10 or ≥10 years). The study concluded that screening can be repeated safely at 2-year intervals in any patient without retinopathy.
8. In 2015, Leese [40•] reported results from a Four Nations working group which combined data from screening programmes in England, Scotland, Wales, and Northern Ireland. This was an observational study based on retinal grading results between 2005 and 2012. In total, 354,549 patients were observed for up to 4 years during which 16,196 patients progressed to referable retinopathy. Of patients with no retinopathy in either eye for two successive screening episodes at least 12 months apart, the conditions of between 0.3% (95% CI 0.3–0.8%) and 1.3% (1.0–1.6%) of patients progressed to referable retinopathy, and rates of treatable eye disease were <0.3% at 2 years. The corresponding progression rates for patients with bilateral background retinopathy in successive screening episodes were 13–29% and up to 4%, respectively, in the different programmes.
9. In 2016, Hughes [41] recommended that the optimum screening intervals should be determined from time to active laser treatment. This is a cohort study of ophthalmologic outcomes in unselected diabetic patients attending a community screening programme in 2001/2002. At screening, 2493 had no retinopathy; 424 had mostly minor degrees of non-proliferative retinopathy. Survival analysis showed that very few of the no retinopathy at screening group required laser therapy in the early years compared with the non-proliferative retinopathy group (*p* < 0.001). The study suggested that, based on requirement for laser therapy, the screening interval for diabetic patients with no retinopathy can be extended to 2 to 3 years.

B. Modelling studies of the natural history of DR

1. In 2011, Aspelund [42] reported on the development of a mathematical algorithm based on epidemiological data on risk factors for DR. Through a website, www.risk.is, the algorithm receives clinical data, including type and duration of diabetes, HbA1c or mean blood glucose, blood pressure, and the presence and grade of retinopathy. These data are used to calculate risk for sight-threatening retinopathy for an individual’s worse eye over time. The database for DR at the Department of Ophthalmology, Aarhus University Hospital, Denmark, was used to empirically test the efficacy of the algorithm. The algorithm recommends screening intervals ranging from 6 to 60 months with a mean of 29 months. This is 59% fewer visits than with fixed annual screening. This amounts to 41 annual visits per 100 patients.
2. In 2012, Mehlsen [43], from the same group of researchers, reported on the development of a model using multiple logistic regressions, with previously defined individual risk factors, for individualised determination of the screening interval in DR. The model was tested on 1372 patients screened during year 2000.
3. In 2012, Chalk [44] reported on the development of a simulation model to predict the likely impact of screening patients with type 2 diabetes, who have not been diagnosed with DR, every 2 years rather than annually. The model was populated with data obtained from the annual retinopathy screening in Devon, UK for a population of approximately 20,000 people with diabetes and generated comparative 15-year forecasts to assess the differences between the current and proposed screening policies. The simulation model predicted that implementing a 2-year screening interval for patients with type 2 diabetes without evidence of DR does not increase their risk of vision loss, and this policy could reduce screening costs by ~25%.
4. In 2013, Stratton [45••] described a risk stratification for time to development of sight-threatening DR which included results from two consecutive screening episodes.
5. In 2013, Looker [46] described the predicted impact of extending the screening interval for DR: the Scottish Diabetic Retinopathy Screening programme The lowest probability for transitioning to referable background or proliferative retinopathy was among people with two consecutive screens showing no visible retinopathy, where the probability was <0.3% for type 1 and <0.2% for type 2 diabetes at 2 years. The study concluded that transition rates to referable diabetic eye disease were lowest among people with type 2 diabetes and two consecutive screens showing no visible retinopathy. If such people had been offered two yearly screening, the Drag Reduction System (DRS) would have needed to screen 40% fewer people in 2009.
6. In 2014, Day [47] reported the sensitivity of DR-associated vision loss to screening interval in an agent-based/discrete event simulation model to examine the effect of changes to screening interval on the incidence of vision loss in a simulated cohort of veterans with DR. DR-associated vision loss increased as the screening interval was extended from 1 to 5 years (*p* < 0.0001). This increase was concentrated in the third year of the screening interval (*p* < 0.01). There was no increase in vision loss associated with increasing the screening interval from 1 to 2 years (*p* = 0.98).
7. In 2016, Lund [48] reported a study to validate a mathematical algorithm [42] that calculates the risk of DR progression in a diabetic population with the UK staging (R0–3; M1) of DR. A cohort of 9690 individuals with diabetes in England was followed for 2 years. The algorithms calculated the individual risk for development of pre-proliferative retinopathy (R2), active proliferative retinopathy (R3A), and diabetic maculopathy (M1) based on clinical data. The algorithm predicts the occurrence of the given DR stages with area under the curve = 80% for patients with type 2 diabetes (CI 0.78 to 0.81). Of the cohort, 64% is at less than 5% risk of progression to R2, R3A, or M1 within 2 years. By applying a 2-year ceiling to the screening interval, patients with type 2 diabetes are screened on average every 20 months, which is a 40% reduction in frequency compared with annual screening.

C. Cost-effectiveness studies

1. In 2000, Vijan [49] examined the marginal cost-effectiveness of various screening intervals for eye disease in patients with type 2 diabetes, stratified by age and level of glycaemic control using a Markov cost-effectiveness model. The study concluded that annual retinal screening for all patients with type 2 diabetes without previously detected retinopathy may not be warranted on the basis of cost-effectiveness, and tailoring recommendations to individual circumstances may be preferable. Patients’ medical risk factors such as poor control were found to have a substantially higher risk and extending intervals without regard for medical risk factors may not be warranted in all settings.
2. In 2015, Scanlon [50••] reported on the development of a cost-effectiveness model for optimisation of the screening interval in DR screening (funded by the HTA programme). Risk factors were identified in Gloucestershire, UK using survival modelling. Two personalised risk stratification models were employed: two screening episodes (SEs) (low, medium, or high risk) or one SE with clinical information (low, medium-low, medium-high, or high risk). The risk factor models were validated in other populations in Nottinghamshire, South London, and East Anglia (all UK). Data were obtained in Gloucestershire from 12,790 people with diabetes with known risk factors to derive the risk estimation models, from 15,877 people to inform the uptake of screening and from 17,043 people to inform the healthcare resource-usage costs. Two stratification models were developed: one using only results from previous screening events and one using previous screening and some commonly available GP data. Both models were capable of differentiating groups at low and high risk of development of sight-threatening DR. In this study, the people with no DR in either eye were found to be at the low risk of progression to sight-threatening DR over a 2-year period (event rate 4.8 per 1000 person years). Using either risk stratification models, screening patients at low risk every 5 years was the most cost-effective option, with a probability of 99–100% at a pound 30,000 per quality-adjusted life-year threshold. For the medium-risk groups, screening every 3 years had a probability of 43–48%, while screening high-risk groups every 2 years was cost-effective with a probability of 55–59%.

D. Commentaries

1. A commentary in 2003 by Professor Ron Klein [51] on the recommendations from the Younis studies [32, 33] made the following points:

- Long intervals between follow-up visits may lead to difficulties in maintaining contact with patients and may give patients the impression that vision loss is unlikely and therefore not a concern.
- That it might be better to have a conservative guideline of yearly examinations with deviations based on evaluation of risk (glycemic and blood pressure control) rather than having a uniformly long interval.
- The ability to generalise the observations of the Liverpool study to other screening situations will depend on the comparability of the population of people with diabetes being screened to those in the Liverpool study and the sensitivity of the approaches used to detect sight-threatening DR and other ocular conditions.

2. In 2013, Leese [52] wrote a commentary exploring the evidence for moving towards a biennial retinal screening programme for patients with type 2 diabetes and diabetes duration of less than 10 years. He also explained that a UK-Four Nations group was critically looking at the evidence for any such changes.

E. Studies of patient behavior or opinions of extending the screening interval

In 2012, Yeo conducted two studies [53, 54]:

- The first [53] was based on 1550 questionnaires distributed at DR clinics in Wales, with 600 complete responses analysed. Eighty-five percent (*n* = 507) felt that they should have their eyes screened every year. However, 65% (*n* = 390) of respondents would accept screening at 2- or 3-year intervals if medical evidence showed that it was safe.
- The second [54] was based on a response rate of 86.4% from the 198 questionnaires administered at clinics, which included a discrete choice experiment contained eight pairwise choices in which screening provision was described by five attributes: frequency of screening, travel time, results time, ability of screening to detect other changes, and explanation of results. Data were analysed using logistic regression techniques. Respondents valued four out of the five attributes [ability of screening to detect other changes (*P* < 0.001), explanation of results (*P* = 0.02), frequency of screening (*P* < 0.001), and travel time (*P* = 0.007)]. Results time was not significant (*P* = 0.1). The study concluded that respondents were willing to accept a longer screening interval, as long as preferences for other attributes of service provision (ability of screening to detect other changes, explanation of results and travel time) were made available.

F. Review articles on screening intervals

1. In 2013, Echouffo-Tcheugui [55] published a systematic review of screening intervals for DR and incidence of visual loss and included studies from PubMed and EMBASE databases which were searched until December 2012. Analysis of 15 studies showed that the aggregated evidence from both the natural history and cost-effectiveness models favors a screening interval > 1 year, but ≤ 2 years. Such an interval would be appropriate, safe, and cost-effective for people with no DR at diagnosis, while screening intervals ≤ 1 year would be preferable for people with pre-existing DR. A 2-year screening interval for people with no sight-threatening DR at diagnosis may be safely adopted. For patients with pre-existing DR, a shorter interval ≤ 1 year is warranted.
2. In 2016, Taylor-Phillips [56] conducted a systematic review to determine whether it was safe to recommend that the DR screening interval in the UK could be extended to beyond 1 year. Electronic searches were performed on 1 October 2013, and hence, no studies after this date would have been included. The study concluded that there was insufficient evidence at that time to recommend a move to extend the screening interval beyond 1 year.
3. In 2016, the UK National Screening Committee reviewed [57] the evidence available. They recommended a change from 1- to 2-year screening intervals for people at a low risk of sight loss. The definition of low risk is two successive diabetic eye screening appointments with photographic grading of no DR. They recommended that the current annual screening interval should remain for all those with mild retinopathy detected in either eye. They provided four appendices as evidence, which included work by the groups that had previously published [40•, 56]. They also provided a supplementary literature review on ‘Does a change in screening interval lead to a subsequent change in uptake?’ This review was unable to find sufficient evidence to support the notion that a change in screening interval would result in a change in uptake of a screening programme. There was also an assessment by UK Department of Health Economists that is available on the website, using a cost-utility approach, whether it is cost-effective to change screening intervals, within the NSC diabetic eye screening programme, according to patient risk, using pre-publication results from Health Economists at Oxford University who had worked on the HTA project—Scanlon [50••].

## Conclusions

The data from real-world screening programmes has demonstrated that people with diabetes who have no DR in either eye are at the low risk of progression to referable or sight-threatening DR over a 2-year period (event rate 4.8 per 1000 person years). Low event rates appear to be irrespective of whether the diagnosis of no DR is based on one-field non-mydriatic photography as is used in the protocol in Scotland [46] or northern Spain [36] or whether it is based on 2-field mydriatic digital photography as is used in other UK countries [32, 33, 35] and Sweden [37]. Data was combined between these methods in the Four Nations Study [40•].

It is important to recognise, as pointed out by Professor Klein in his 2003 article [51] that the generalizability of these observations to other screening situations will depend on the comparability of the population of people with diabetes being screened and the sensitivity of the approaches used to detect sight-threatening DR and other ocular conditions.

Cost-effectiveness studies [49, 50••] have suggested that annual screening for all people with diabetes may no longer be cost-effective.

There have been suggestions that the screening interval should be lengthened to 2 years for ‘low risk groups’ which has been variably defined by the following:

1. Two screening episodes with individualised risk factor data
2. Two screening episodes with no retinopathy
3. One screening episode with individualised risk factor data

The only study that compared area under the curve of the receiver operator curves for these three groups was the Health Technology Assessment study by Scanlon [50••] which found the following:

1. Two screening episodes with individualised risk factor data—AUC 0.786 (95% CI 0.759 to 0.813)
2. Two screening episodes with no retinopathy—AUC 0.759 (95% CI 0.732–0.788)
3. One screening episode with individualised risk factor data—AUC 0.774 (95% CI 0.748–0.800)

In order to add in the risk factor data to screening data, there needs to be links available to this extra data that are not readily available to many population-based screening programmes. Hence, the UK National Screening Committee has recommended a change from 1- to 2-year screening intervals for people who have two successive diabetic eye screening appointments with photographic grading of no DR.

There is a large gap in knowledge and research on how this will affect individual patient behaviors and attendance for screening programmes. Work by Yeo [53, 54] has suggested that respondents would accept screening at 2- or 3-year intervals if medical evidence showed that it was safe and as long as preferences for other attributes of service provision (ability of screening to detect other changes, explanation of results and travel time) were made available. However, there is no current evidence of patient’s behavior in these circumstances. There is an on-going concern that if patients are told that they are at the low risk of progression that:

1. They may make less effort in the control of their diabetes and hence put themselves at a greater risk.
2. They may be less likely to attend in the future.

A currently funded project in Liverpool, UK [58] is studying the introduction of personalised screening intervals and may help to answer some of these questions in similar populations.
